# Genomic Analysis of Lymphoma Risk in Bullmastiff Dogs

**DOI:** 10.3390/vetsci10120703

**Published:** 2023-12-14

**Authors:** Sally A. Mortlock, Monica C. Asada, Pamela Xing Yi Soh, Wei-Tse Hsu, Carol Lee, Peter F. Bennett, Rosanne M. Taylor, Mehar S. Khatkar, Peter Williamson

**Affiliations:** 1 Sydney School of Veterinary Science, Faculty of Science, The University of Sydney, Camperdown, NSW 2006, Australiamehar.khatkar@sydney.edu.au (M.S.K.); 2 School of Life and Environmental Sciences, Faculty of Science, The University of Sydney, Camperdown, NSW 2006, Australia; 3 School of Medical Sciences, Faculty of Medicine and Health, The University of Sydney, Camperdown, NSW 2006, Australia; 4 School of Animal and Veterinary Sciences, The University of Adelaide, Roseworthy, SA 5371, Australia

**Keywords:** canine, lymphoma, GWAS, Bullmastiff

## Abstract

**Simple Summary:**

Lymphoma is the most common cancer affecting dogs and is particularly prevalent in the Bullmastiff breed. We know that it tends to be inherited, but not in a simple pattern. In this study, we looked at the relatedness of dogs that developed lymphoma amongst 330 pure-bred Bullmastiffs. We also examined their DNA to identify which regions in their chromosomes were more common in the dogs diagnosed with lymphoma. Using a series of statistical tests, we found small segments on two chromosomes that were most interesting. When we examined these regions of DNA in canine databases, we found that they contained genes that are known to play an important role in controlling the growth and development of cells. Further experiments are needed to understand whether these genes have an influence on the development of lymphoma in Bullmastiff dogs; meanwhile, we can test whether our DNA analysis will correlate with other dogs developing lymphoma in their lifetime. The eventual goal is to reduce the burden of cancer and improve health in Bullmastiffs and other breeds.

**Abstract:**

Lymphoma is the most common haematological malignancy affecting dogs and has a high incidence in the Bullmastiff breed. The aim of this study was to identify risk loci predisposing this breed to the disease. The average age of lymphoma diagnosis in 55 cases was less than 6 years, similar to the median age of 64 cases from our clinical and pathology databases. When fine-scale population structure was explored using NETVIEW, cases were distributed throughout an extended pedigree. When genotyped cases (*n* = 49) and dogs from the control group (*n* = 281) were compared in a genome-wide association analysis of lymphoma risk, the most prominent associated regions were detected on CFA13 and CFA33. The top SNPs in a 5.4 Mb region on CFA13 were significant at a chromosome-wide level, and the region was fine-mapped to ~1.2 Mb (CFA13: 25.2–26.4 Mb; CanFam3.1) with four potential functional candidates, including the MYC proto-oncogene bHLH transcription factor *(MYC)* and a region syntenic with the human and mouse lncRNA Pvt1 oncogene (*PVT1)*. A 380 Kb associated region at CFA33: 7.7–8.1 Mb contained the coding sequence for SUMO specific peptidase7 *(SENP7)* and NFK inhibitor zeta *(NFKBIZ)* genes. These genes have annotations related to cancer, amongst others, and both have functional links to *MYC* regulation. Genomic signatures identified in lymphoma cases suggest that increased risk contributed by the regions identified by GWAS may complement a complex predisposing genetic background.

## 1. Introduction

Lymphoma is the most frequently diagnosed haematological canine cancer, affecting up to 1.2% of the dog population and accounting for 8–20% of cancer cases [1,2,3]. The disease is in many cases treatable but not curable, and resistance to chemotherapy develops with progression of the disease [4,5]. Studies investigating the incidence of lymphoma in dogs have identified an association between disease occurrence and breed [6,7,8,9]. The Bullmastiff breed is one of a number known to have a very high incidence of lymphoma, suggesting that there may be a genetic predisposition to the disease [3,9,10,11,12,13]. Originally bred as estate guardians in Britain in the mid-19th century, the Bullmastiff is a large working breed originally derived from the Bulldog and Mastiff breeds and recognised as pure-bred by The Kennel Club in 1924 [14]. Analysis of lymphoma cases in UK dogs revealed that the incidence of lymphoma was greatest in Bullmastiffs [11,15]. Similar findings have been reported in other studies that found this breed to be at high risk [3,9], with an incidence of lymphoma almost five times the mixed-breed population in American dogs. The diagnosis of lymphoma across all breeds peaks between 7 and 10 years of age, but in Bullmastiffs, many cases present in dogs aged 4–6 years [3,11], and the disease contributes significantly to mortality in young dogs, influencing longevity in the breed as a whole. 

While epidemiology, diagnosis, treatment, and the identification of somatic changes during tumour development have been a focus of much research in canine lymphoma [4,11,12,16,17,18], the complex genetic basis of breed-specific risk factors is still emerging. One study investigated cancer risk loci in Golden Retrievers and identified shared predisposing loci for B-cell lymphoma and haemangiosarcoma in this breed. In other breeds, associations have been described at other loci [19,20,21,22,23,24,25]. The presence of these or other risk loci in the Bullmastiff breed is yet to be confirmed. 

The aim of the present study was to identify risk loci for lymphoma in an Australian Bullmastiff subpopulation. A genome-wide association study (GWAS) was employed to locate genomic regions associated with the disease across all ages and was complemented by a focused analysis of dogs affected at a young age. 

## 2. Materials and Methods

### 2.1. Inclusion of Dogs 

A total of 330 Bullmastiff dogs were included in the DNA study, including 49 lymphoma-affected dogs. Dogs were classified as lymphoma cases based on a standard minimal criterion of fine needle aspirate cytology following clinical diagnosis by a veterinarian, confirmed through a review of a veterinary report, direct contact with the attending veterinarian, or owner communication of a clinical diagnosis. Unaffected dogs included as controls had no clinical evidence of the disease at the time of their inclusion or previously. All dogs were privately owned pets volunteered by owners with informed consent. The study was approved by The University of Sydney Animal Ethics Committee under protocols 4949 and 6013. 

Pedigree data were available through an extended database of registered Bullmastiff dogs [26]. Information regarding lymphoma cases and pedigree backgrounds were provided with consent through relevant veterinarians or reported by owners. The R packages “kinship2”, “pedigree”, and “FamAgg” [27,28,29] were used to assemble pedigrees. 

### 2.2. DNA Extraction and Genotyping

Genotyping was performed for all dogs using genetic material extracted from whole blood or, for a small number of animals, from semen samples. Genomic DNA was extracted from whole blood samples with a standard procedure using the manufacturer’s recommendations (QIAamp DNA Blood Kit Qiagen, Melbourne, Vic, Australia). Genomic DNA from frozen stored semen samples derived from seven dogs was isolated using a modified phenol/chloroform extraction procedure as described previously [26]. Genome-wide SNP genotyping was performed for 220,000 SNPs using the CanineHD BeadChip (Illumina, San Diego, CA, USA; Geneseek, Lincoln, NE, USA). For some cases genotyped using the 170K chip, genotypes were imputed to the 220K chip using BEAGLEv5.1 [30] as described previously [24,31].

### 2.3. Relationship Networks (NetView) 

Relationship networks were constructed as described previously using “netview” and “GGally” implemented in R. using a clustering *k*-value of 10 [32]. Additionally, the package “network D3” https://cran.r-project.org/web/packages/networkD3/networkD3.pdf was employed (accessed on 17 June 2021). Networks were based on either pedigree kinship matrix or by using a distance matrix created in PLINK v1.9 [33] and genotypes for dogs included in the GWAS analysis. 

### 2.4. Genome-Wide Association Analysis 

The process for association analysis has been described previously [24]. Briefly, Genome-wide Complex Trait Analysis (GCTA) [34] software was used to exclude SNPs in the imputed data minor allele frequencies less than 0.02, and to generate genetic relationship matrices (GRMs) for analysis with mixed linear model analysis (MLMA). Plots were generated using R v3.6.1 (R Development Core Team 2011). The Storr method for estimating false discovery rates (q-values) was used on genome-wide and chromosome-wise *p*-values through the R package “qvalue” using the MLMA output [35]. The R package “qqman” was employed to represent the association analysis output [36]. Regional association plots were generated with code modified from R packages “RACER” and “IntAssocPlot” [37,38]. Linkage disequilibrium (LD) values (r^2^) used in the regional association plots were calculated in PLINK v1.9 [33], and gene names were according to NCBI-Entrez.

### 2.5. Haplotype Analysis

Haplotypes were generated in PLINK using a sliding window approach [39]. The hap-window function was used to specify all haplotypes in sliding windows between 2 and 8 SNPs in size, sliding 1 SNP at a time. Haplotype blocks were defined if 95% of informative comparisons between SNPs were in strong linkage disequilibrium (LD) [40]. Fine-scale examination of individual genotypes was also performed manually on aligned genotype data aided by color-coding using the conditional formatting function in Excel.

### 2.6. Composite Selection Signals (CSS) Analysis

Composite selection signals (CSS) analysis was used to evaluate genomic signals in the lymphoma dogs used in the study when compared to dogs at low risk of lymphoma. Details of the CSS method have been described previously [41]. Briefly, CSS combines three different test statistics (F*_st_*, ∆DAF, and XP-EHH). Univariate statistics were converted to fractional ranks between 0 and 1 by 1/(n + 1) to n/(n + 1), where n is the number of SNPs. The inverse normal cumulative distribution function (CDF) was used to obtain the Z-scores from fractional ranks. Averages of Z-scores for each SNP were generated from all statistical tests and the mean Z-scores transferred to *p*-values by normal distribution *N*(0, m^−1^), where m is the number of test statistics. The values of CSS were defined as the logarithmic of *p*-values (−log_10_ of *p*-values). A 1 Mb window was used at each SNP (0.5 Kb on either side), and the means of CSS values in each window were obtained as the smoothed CSS. Signals that exceeded the 99.5% threshold (top 0.005 fraction) of smoothed CSS scores were considered significant regions. The mean value of all significant SNPs within a region were averaged and designated as the regional average CSS value.

### 2.7. DNA Sequencing 

Three previously genotyped individuals were selected for DNA sequencing, including one affected dog, its dam, and an unrelated dog from the control group. Sequencing was performed using 250 bp paired-end reads on the Illumina HiSeq2500 platform. An average of 10x coverage was generated as previously described [42]. Quality control filtering of reads, alignment to CanFam3.1, and variant detection were performed using CLC Genomics Workbench 8.5.1 (CLC bio, Aarhus, Denmark). The workbench was also used to identify variants in sequence categorised as predicted splice sites, amino acid substitutions, and potential changes to protein structure. The effects of each variant were predicted using Variant Effect Predictor (VEP) [43]. 

## 3. Results

### 3.1. Lymphoma Cases

**Figure 1:**
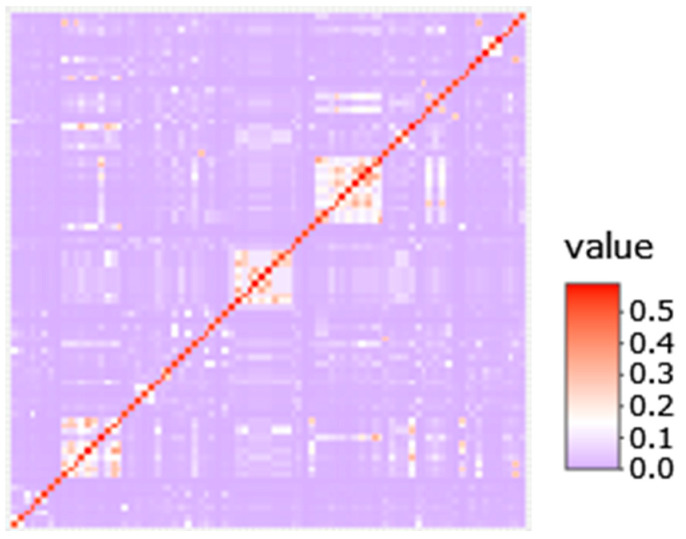
Pedigree relationship matrix of lymphoma cases.

A total of 83 Bullmastiff lymphoma cases were identified; 55 were reported from 273 responses to a preliminary survey, and the remaining cases were reported as new cases or volunteered from owners of deceased animals. A relationship matrix constructed from pedigree data from these dogs is represented in Figure 1. Within the pedigree, 21 cases were progeny traced to a common ancestor and 18 cases to another. 

**Figure 2:**
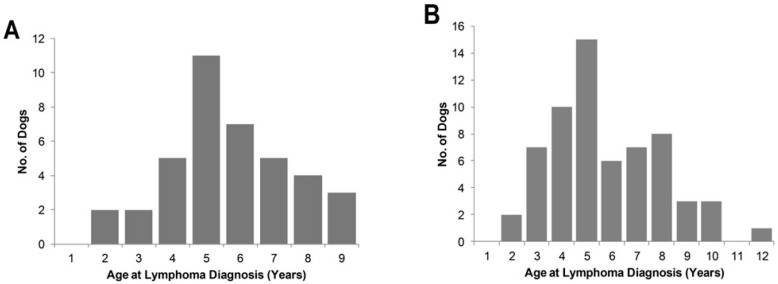
Age distribution of lymphoma cases of (**A**) this study and (**B**) data collected through clinical and pathology databases.

Amongst those with reported age of diagnosis, the average age was 5.7 years, with 69% of cases in dogs six years or younger (Figure 2A). This age profile was similar to data collected through clinical and pathology databases, in which 64 cases of Bullmastiff lymphoma were identified. The average age of diagnosis for these cases was 6.62 years (range 3 to 12.5 years) with a median of 6 years (Figure 2B). The anatomical distribution of lymphoma was partially recorded for 98% of cases; the most common reported was nodal lymphoma (80%), followed by systemic (16%) and epitheliotropic (4%). A large proportion the cases reported from clinical and pathology sources were not subtyped (83%); of 11 cases that were, 8 (73%) were B-cell malignancies and 3 (27%) were T-cell. 

### 3.2. Genome-Wide Association Analysis

**Figure 3:**
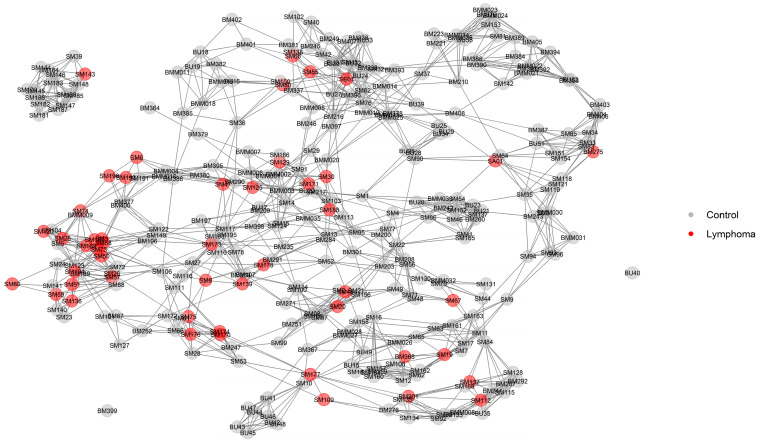
Relationship network based on genotypes of cases and control group dogs.

After the imputation and filtering of the genotype data, 330 dogs (49 lymphoma cases and 281 controls) and 153,900 SNPs were included for genomic analyses. Dogs classified as lymphoma cases were mapped onto a high-definition relationship network diagram produced from all genotyped dogs using NETVIEW (Figure 3). Cases were distributed across the network, though two subpopulation clusters were free of cases. One smaller cluster was not connected to the main network with these parameter settings but was connected to one case. 

**Figure 4:**
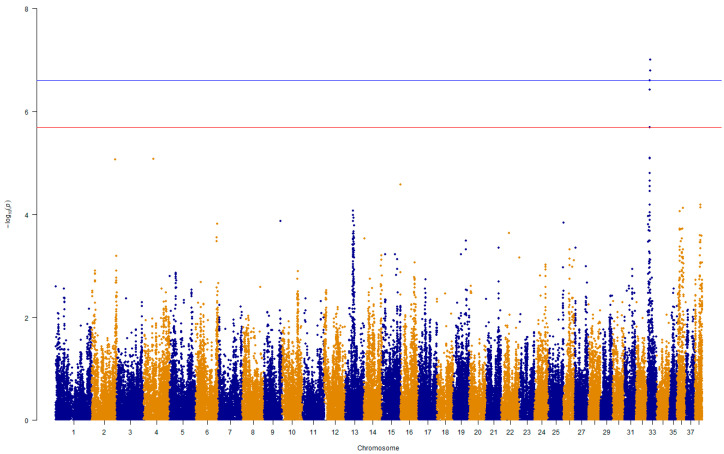
Manhattan plot of genome-wide SNP associations in lymphoma cases compared to the control group dogs. Threshold values represent q-values of 0.01 (blue) and 0.05 (red).

Association analysis revealed a genome-wide significant signal on chromosome 33 (Figure 4). Amongst the 100 SNPs ranked by *p*-value, 34% were on CFA33, 36% on CFA13, 13% on CFA36, and 12% on CFA38 (Table S1). There were two singletons on CFA2 and 4. Chromosome-wise analysis showed significant SNPs in regions on CFA13 and two regions on CFA36 and CFA38 (q-value < 0.01) in addition to the region on CFA33 (Figure S1).

Analysis of clinical data showed a significant number of Bullmastiff dogs that were diagnosed with lymphoma at a young age. We conducted a GWAS on a subpopulation of 111 dogs consisting of cases diagnosed at age 6 years or less (*n* = 23) and a control group of dogs that remained free of disease and were above the age of 7 years (*n* = 88). The most prominent association signals were confirmed on CFA13 and CFA33. When ranked by *p*-value, the top 3 were on CFA33, while 26 out of the top 30 SNPs were on CFA13 (Table S2). Only the top SNP reached genome-wide significance following adjustment, but 17 SNPs on CFA13 and 21 SNPs on CFA33 reached chromosome-wide significance (q-value < 0.05). There were no significant SNPs on CFA36 or 38 in this analysis.

**Figure 5:**
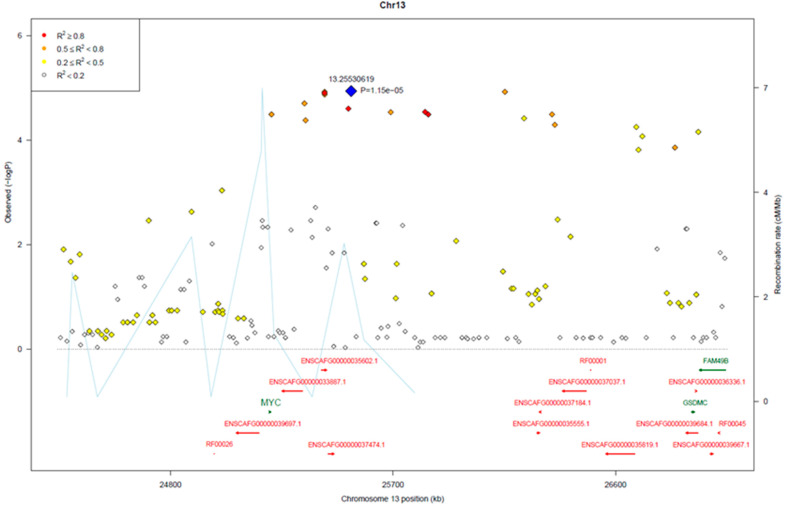
Regional plot of associated SNPs on CFA13.

The top-ranked SNPs on CFA13 were located within a 5.4 Mb region (~24.4–29.8 Mb). Details of this region are shown in Figure 5. The top associated SNPs were in strong linkage disequilibrium with the top-ranked SNP (r^2^ > 0.8), potentially indicating one underlying causal variant. Examination of genotypes revealed short runs of homozygosity within the region that were prevalent in the affected dogs. The most frequent haplotypes and homozygous SNPs in the affected dogs were located within a 1.2 Mb region from 25.2 to 26.4 Mb on CFA13. One region defined by eight SNPs (13.25196069–13.25278832 bp; GCGCGGAA) spanned the proto-oncogene *MYC* and nearby predicted precursor sequence for *miR-1204*. Another two homozygous regions, beginning at SNPs 13.25299888 and 13.25423168, flanked the predicted precursor sequence for *miR-1205* and *miR-1206* and covered the sequence that is syntenic with human *PVT1*. 

**Figure 6:**
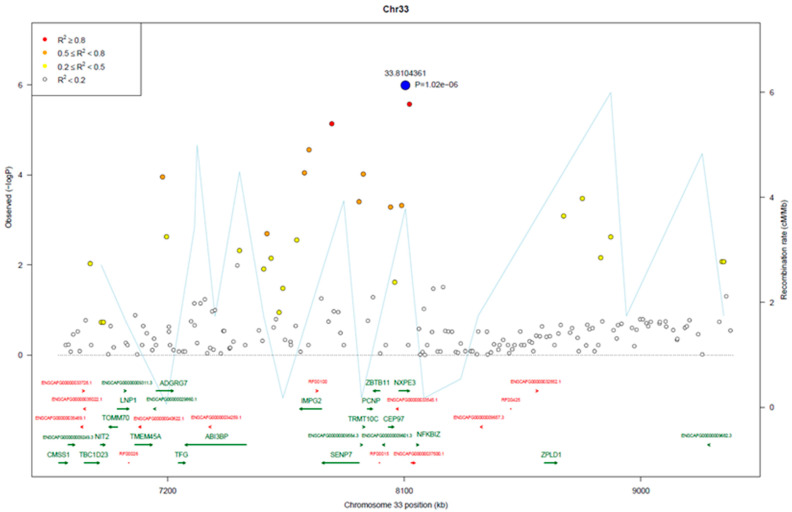
Regional plot of associated SNPs on CFA33.

The most significant SNPs on CFA33 were within a 3.8 Mb region (7.7–8.1 Mb) with strong LD between SNPs at the regional peak (r^2^ > 0.8) (Figure 6). Candidate gene analysis within this region showed SNP 33.7824270 was within the *SENP7* gene, whereas SNPs 33.8104361 and 33.8119663 were closely linked to *NFKBIZ*. Both genes have annotations related to cancer and both have functional links to *MYC* regulation. Interestingly, heterozygous genotypes across the three SNPs were significantly higher in the cases than the control samples. Homozygosity for the top three SNPs was 60% (52/88) in the control dogs compared to 13% (3/23) of cases (*p*-value = 0.0012). 

### 3.3. Genomic Signatures in Lymphoma-Affected Bullmastiff Dogs

Founder effects and selection have a very strong influence on the background genotypes of pure-breed dogs. The consequences of these effects on variation in the genome means that some regions are not detected by within-breed GWAS analysis. To map regions in the lymphoma-affected Bullmastiff dogs in this study, we compared the genotypes of the dogs included in this study to 498 dogs from 26 breeds that have a low incidence of lymphoma (Table S3). The top-ranked regions (0.1%) were identified on CFA1, 3, 5, 7, 10, 18 and 32. The locations of the identified regions are listed in Table S4. Additional regions were identified at a 0.5% threshold on CFA1, 3, 7, 8, 20, 22, 26, 29 and 30 (Table S4). 

### 3.4. DNA Sequence Variants

Focusing on the associated CFA13 5.4 Mb region identified in the GWAS, variants were filtered to remove any that did not follow a recessive pattern for the disease-associated genotypes in cases and controls. A total of 1869 variants were identified from the sequencing data that were homozygous in the affected dogs and followed the pattern of the risk-associated genotypes. Variants were annotated against exons and known variants. Of the variants in the region, 1500 were intergenic and 369 gene-coding, 8 of which were located in exons, including 1 insertion and 7 SNPs. One non-synonymous variant was found in *GSDMC* and two in *LDH*; each had predicted moderate impacts and were coded as “tolerated” using SIFT. Additionally, 1 gene-coding variant was located within the 1.2 Mb region, with the remaining 380 variants falling into intergenic regions. While there were no non-synonymous variants within the 1.2 Mb region, a putative insertion was identified in an AT-rich region of the 3′ UTR of *MYC.* The variant list is summarized in Table 1. Based on the sequence analysis, we analysed eight variants using PCR typing across the region, which encompassed six genes: *Myc*, *GSDMC*, *LDH*, *TMEME71*, *WISP1*, and *NDRG1*. There was no further improvement in the segregation of cases when compared to the original risk alleles. 

## 4. Discussion

Lymphoma causes substantial morbidity and mortality in dogs. One study across 72 breeds found that an average of 27% of deaths recorded were from cancer, and the most common form of cancer in dogs was lymphoma [15]. Analysis of a subpopulation of Australian Bullmastiff dogs revealed the average age of death as 6.9, which was comparable to that reported in a study of longevity in pure-breed dogs, in which Bullmastiffs in the UK had an average age of death of 7.5 years [15]. Cancer in UK Bullmastiffs accounts for 37.5% of deaths [13,15]. Within the dogs reported to have been diagnosed with lymphoma in the present study, a significant number were in younger dogs. Previous studies have found that the incidence of lymphoma in all dogs increases with age, with the average age of onset between studies ranging from 6.7 to 9.0 and a peak occurring at 10 years [11,44]. The average age of lymphoma diagnosis reported in our study was 5.7 years, and although there is always a risk of ascertainment bias when collecting data focused on a specific disease, it was concordant with a similar median age of diagnosis at 6 years revealed in the clinical and pathology databases. Again, this is comparable to the reported age of diagnosis in UK Bullmastiffs, which was significantly higher between the ages of 4 and 6 years. Though there were only a few cases with the reported subtype in the present study, the predominance of B-cell lymphoma (73%) compared to T-cell (27%) is similar to that reported for mastiff breeds (68% B-cell and 32% T-cell) [7]. 

The patterns of disease and its distribution across dog breeds have been key indicators that canine lymphoma has a genetic component. The analysis presented here showed that lymphoma in Bullmastiffs is associated with at least two genomic regions, one each on CFA13 and CFA33. 

A 1.2 Mb region within a larger 5.4 Mb-associated region on CFA13 was enriched for homozygous genotypes within the cases. We selected a small number (n = 3) of representative samples for DNA sequence analysis. This trio of samples was representative of the possible genotypes within the associated CFA13 region: one was from a lymphoma-affected offspring of another and the third dog was an unaffected individual; SNP genotyping confirmed the lymphoma-affected dog was homozygous for the CFA13 risk haplotype, the parent dog was heterozygous for the CFA13 risk haplotype, and the unrelated dog was homozygous for the non-risk CFA13 haplotype. Amongst other variants, we found an insertion in the 3′UTR of MYC that was of potential interest, but this requires additional study to understand any significance. The 1.2 Mb region spanned the *MYC* proto-oncogene and nearby precursor sequences for *miR-1204*, *miR-1205*, and *miR-1206* in a region syntenic to human *PVT1* [45], which has ontologies relating to cell growth, differentiation, and cancer [46,47,48]. Variants in this region have been associated with diffuse large B-cell lymphoma (DLBCL) and follicular lymphoma risk in humans [49]. *PVT1* is annotated as a long non-coding RNA (lncRNA) and belongs to a large family of lncRNA that have been shown to have differential expression profiles in DLBCL in humans and dogs. They may also be regions that harbour smaller miRNAs, as is the case for PVT1, which contains a total of six miRNAs. It has been recognised as a site of retroviral insertions, reciprocal translocations, and amplifications in tumours, and has a regulatory role in lymphomagenesis and lymphocyte activation [46,48]. Indeed, the functional interactions of *MYC* and *PVT1* appear to be critical for an effect of this region [50], and may involve a complex extrachromosomal mechanism by which PVT1 amplifies the proliferative effects of oncogenes [51]. Like most miRNAs, the miRNAs 1204, -1205, and -1206 have complex biology and the potential to influence expression of a wide range of genes. According to miR databases (https://maayanlab.cloud/Harmonizome (accessed on 5 December 2023), miR-1204 may affect up to 135 target genes, miR-1205 may affect 688 targets, and miR 1206 may affect 378 targets. It is also predicted that miRNAs in this region influence the transcript levels of *MYC,* either directly or through upstream activation sites [46]. Supporting observations were also seen, which suggested that *PVT1* acts to stabilize the Myc protein [46]. We did not find any variation in *MYC* gene expression in a subset of this group of dogs in a previous study [52,53], but preliminary observations on a very small number of lymphoma-affected lymph nodes suggested that Myc protein levels were affected by genotypes in the CFA13-associated region. Whether this effect is significant would need to be validated in a much larger study.

Amplification of the *MYC-PVT1* region may also contribute to lymphoma cases from other breeds. Copy number gain of CFA13 has been identified as the most recurrent aberration observed in canine lymphomas, occurring in 37–58% of tumours [17,54,55]. Copy number increase of CFA13 is found in both canine B- and T-cell lymphoma, including in samples from Bullmastiff cases [17]. A study using high-resolution array comparative genomic hybridization to identify chromosomal copy number aberrations in 12 dogs with Diffuse Large B-cell Lymphoma found aberrations in the region containing *MYC* in 75% of cases [55]. These findings are analogous to those in human non-Hodgkin lymphomas, where the syntenic chromosomal region at 8q24.21 is commonly amplified [56,57,58,59]. 

There is also a potential interaction with the MYC functional network and genes from the associated region on CFA33. Genes found within this region included *SENP7* and *NFKBIZ*. Both are known to intersect with the regulation of MYC. Mutations of *SENP7* and *NFKBIZ* have been reported in malignancies in humans [60,61]. *SENP7* is a small Ubiquitin-like modifier (SUMO) protease that regulates MYC by post-translational modification. SUMO proteins are involved in Wnt/β-catenin pathways and potentially in cell cycle progression and chromosomal segregation [62]. IκBζ, the protein encoded by *NFKBIZ*, is an ankyrin-repeat protein with a similar function to other NFκB regulatory inhibitor proteins. These act as nuclear regulators of NF transcriptional binding sites. NFκB pathway activation is a feature of many lymphomas, and *NFKBIZ* expression is a key hallmark of the activated subtype of DLBCL in humans, which is similar to the most common form in dogs [63,64,65,66,67,68].

The associated regions found in this study add to a list of different regions found to be associated with lymphoma or other haematological malignancies in various breeds [20,21,22,23,24,25]. These regions appear to be specific to individual breeds, although there may be some degree of pleiotropy [22]. Lymphoma is a diverse set of cancers that share the common lymphocytic origin. This diversity may explain why the associated regions described to date cannot account for all lymphoma cases within the respective studies, which may be expected for complex disease analysis. In our study, we included all reported cases of lymphoma as defined by generic criteria. Therefore, our results reflect an analysis of overall risk in the Bullmastiff subpopulation included in the study in which we expect to predominantly see the B-cell subtype. The analysis is subject to the limitations of GWAS and a case–control retrospective design when studying a complex trait. It highlights genes of interest within associated regions but in no way could claim to represent an exhaustive list of genes underlying lymphoma risk. Indeed, it is likely that multiple relevant associations are not detected by GWAS using SNP genotyping and common undetected variants within a breed or clade may contribute to the phenotype. In the Bullmastiff dogs in this study and in previous studies [69,70], we identified genomic signatures that have the potential to contribute to disease risk; for example, a well-known region on CFA26 that is commonly found in mastiff breeds contains candidate genes that have been implicated in cancer risk [71]. Many of these regions have complex functional annotations, and unravelling their role in disease may benefit from current efforts towards large-scale sequence analysis of the canine genome and eventual detailed functional validation [72,73]. 

## 5. Conclusions

Like all retrospective studies based on case–control GWAS analysis, this study was limited by the number of lymphoma cases and by the possibility of ascertainment bias. However, the results of the analysis demonstrate that lymphoma in Bullmastiff dogs is associated with at least two genomic regions on CFA13 and CFA33. Both regions contain candidate genes with known lymphoma-associated annotations which are linked to the oncogenic effects of the *MYC* gene, amongst other functions. Other regions identified as genomic signatures in the lymphoma-affected subgroup are potential contributors to the overall lymphoma risk profile. 

## Figures and Tables

**Table 1:** DNA sequence variants in CFA13 lymphoma risk-associated region. Homozygous variants located within exons and UTRs that are within the 5.4 Mb region of CFA13 associated with lymphoma cases.

Location	Type	Ref	Allele	Gene	Exon Number	Consequence	A.A. Change
25205559^ 25205560	Ins	-	T	MYC	3/3	3 prime UTR	-
26907214	SNP	A	G	GSDMC	1/12	missense	H/R
28304328	SNP	G	A	LDH	1/1	missense	D/N
28304341	SNP	T	C	LDH	1/1	missense	V/A
29249168	SNP	C	T	TMEM71	1/11	5 prime UTR	-
29681948	SNP	G	A	WISP1	4/4	3 prime UTR	-
29682320	SNP	A	T	WISP1	4/4	3 prime UTR	-
29689113	SNV	A	T	NDRG1	16/16	3 prime UTR	-

## Data Availability

Data used for this study is available within this publication.

## Supplementary Materials

The following supporting information can be downloaded at: https://www.mdpi.com/article/10.3390/vetsci10120703/s1, Figure S1: Manhattan plots of SNP association analyses on CFA13 (A), CFA33 (B), CFA36 (C) and CFA 38 (D); Table S1: Top SNPs ranked by p-value; Table S2: Top associated SNPs in young dogs; Table S3: Breeds used as reference in CSS analysis with low risk of lymphoma. The total number of samples was 498 following SNP quality implementation. Table S4: Genomic regions identified as signatures in Bullmastiff dogs diagnosed with lymphoma. Data Files.
