# Supplementary material for: Genomic Analysis of Lymphoma Risk in Bullmastiff Dogs
Source: Vet Sci. 2023 Dec 14;10(12):703. doi: 10.3390/vetsci10120703 (PMC10747964; doi:10.3390/vetsci10120703)
Supplement: Supplementary file 1 [file vetsci-10-00703-s001.zip › Supplementary Materials.pdf]

Figure S1: Manhattan plots of SNP association analyses on CFA13 (A), CFA33 (B), CFA36 (C) and CFA 38 (D).

A

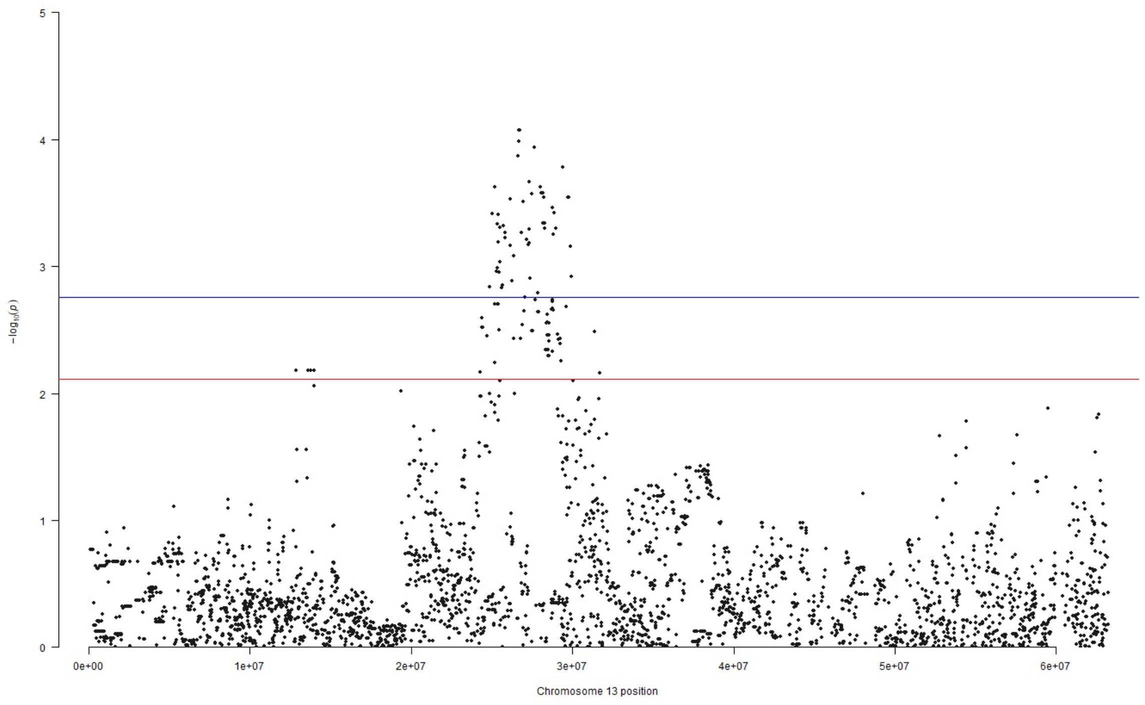

B

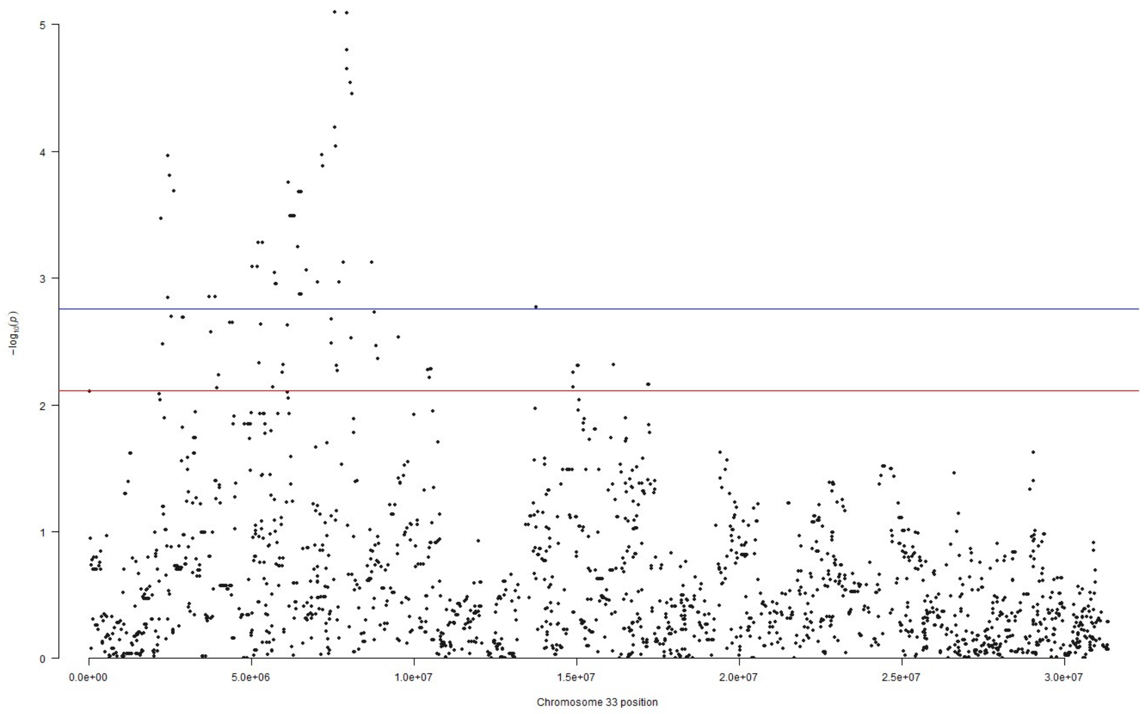

5
4
3
2
1
0
−log10(p)
0.0e+00
5.0e+06
1.0e+07
1.5e+07
2.0e+07
2.5e+07
3.0e+07
Chromosome 33 position

C

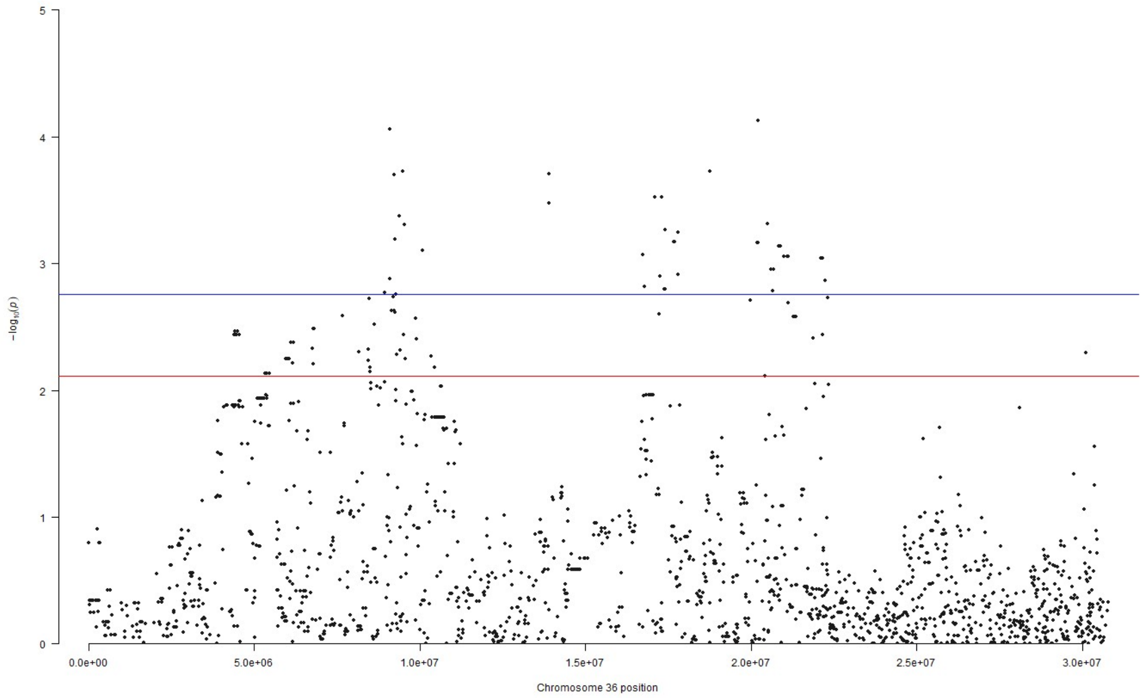
5
4
3
2
1
0
$-\log_{10}(p)$
0.0e+00
5.0e+06
1.0e+07
1.5e+07
2.0e+07
2.5e+07
3.0e+07
Chromosome 36 position

D

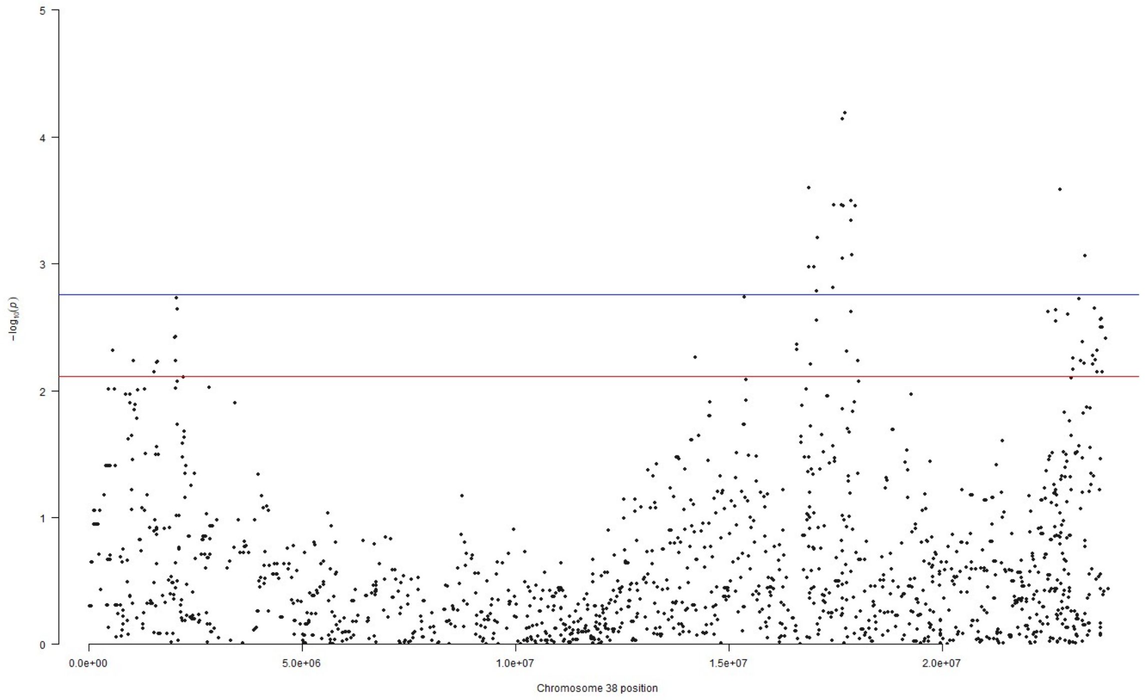

5
4
3
2
1
0
$-\log_{10}(p)$
0.0e+00
5.0e+06
1.0e+07
1.5e+07
2.0e+07
Chromosome 38 position

Table S1: Details of top associated SNPs

CHR	BP	P.value	A1	A2	Freq	b	SE
33	8119663	0.00000	C	A	0.23	0.19	0.04
33	8104361	0.00000	G	A	0.25	0.18	0.03
33	7948722	0.00000	G	A	0.25	0.18	0.03
33	7824270	0.00000	A	G	0.25	0.17	0.03
33	7720211	0.00000	A	G	0.19	0.18	0.04
33	7737742	0.00000	A	G	0.19	0.18	0.04
33	7577636	0.00001	C	A	0.32	0.14	0.03
33	7944060	0.00001	G	A	0.21	0.17	0.04
4	33686301	0.00001	A	G	0.26	0.18	0.04
2	79804014	0.00001	A	C	0.33	0.12	0.03
33	7947940	0.00002	A	C	0.21	0.16	0.04
33	7927920	0.00002	G	A	0.31	0.14	0.03
33	8047765	0.00003	G	C	0.30	0.13	0.03
33	8089901	0.00004	A	G	0.32	0.13	0.03
33	7565433	0.00006	A	G	0.15	0.16	0.04
38	17725938	0.00006	A	G	0.17	0.16	0.04
38	17665758	0.00007	A	G	0.14	0.17	0.04
38	17666204	0.00007	A	G	0.14	0.17	0.04
38	17668116	0.00007	A	G	0.14	0.17	0.04
36	20206887	0.00007	A	G	0.06	0.25	0.06
13	26684157	0.00008	G	A	0.42	-0.12	0.03
13	26708681	0.00008	G	A	0.42	-0.12	0.03
36	9081255	0.00009	G	A	0.22	0.14	0.04
33	7593370	0.00009	G	A	0.26	0.13	0.03
13	26693097	0.00010	G	A	0.42	-0.12	0.03
33	7180525	0.00011	A	G	0.28	0.13	0.03
33	2441102	0.00011	G	A	0.18	0.15	0.04
13	27637942	0.00012	A	G	0.42	0.12	0.03
33	7195565	0.00013	G	A	0.15	0.16	0.04
13	26648648	0.00013	G	A	0.42	-0.12	0.03
33	2477174	0.00016	C	A	0.18	0.15	0.04
13	29380799	0.00016	C	G	0.13	0.16	0.04
13	29383945	0.00016	A	G	0.13	0.16	0.04
13	29393693	0.00016	G	A	0.13	0.16	0.04
13	29395323	0.00016	G	A	0.13	0.16	0.04
13	29404434	0.00016	A	G	0.13	0.16	0.04
33	6142107	0.00018	A	G	0.21	0.14	0.04
36	9482475	0.00019	A	G	0.13	0.17	0.04
36	18758528	0.00019	C	A	0.06	0.23	0.06
36	18766991	0.00019	G	A	0.06	0.23	0.06
36	13909161	0.00020	C	A	0.13	0.16	0.04
36	9216972	0.00020	G	A	0.23	0.13	0.04
33	2626063	0.00020	A	G	0.19	0.14	0.04
33	6452002	0.00021	A	G	0.15	0.15	0.04
33	6467209	0.00021	G	A	0.15	0.15	0.04
33	6506969	0.00021	A	G	0.15	0.15	0.04
33	6531971	0.00021	A	G	0.15	0.15	0.04
13	27331125	0.00022	G	A	0.17	0.15	0.04
13	25208544	0.00023	A	C	0.50	-0.11	0.03
13	28029410	0.00024	G	A	0.46	0.11	0.03
38	16878192	0.00025	A	G	0.21	0.13	0.04

38	22764291	0.00026	A	C	0.08	0.21	0.06
13	28197615	0.00026	A	G	0.46	0.11	0.03
13	28075353	0.00027	G	A	0.46	0.11	0.03
13	27466313	0.00027	A	G	0.48	0.11	0.03
13	29743592	0.00029	G	A	0.48	-0.11	0.03
13	29776513	0.00029	A	G	0.48	-0.11	0.03
13	28223435	0.00029	A	G	0.47	0.11	0.03
13	26152730	0.00029	A	G	0.50	-0.11	0.03
36	17098631	0.00030	G	A	0.07	0.22	0.06
36	17289324	0.00030	A	G	0.07	0.22	0.06
13	26935106	0.00031	A	C	0.42	-0.11	0.03
38	17858516	0.00032	G	A	0.22	0.13	0.04
33	6181465	0.00032	G	A	0.15	0.14	0.04
33	6183488	0.00032	G	A	0.15	0.14	0.04
33	6196948	0.00032	A	G	0.15	0.14	0.04
33	6229975	0.00032	G	A	0.15	0.14	0.04
33	6283136	0.00032	A	G	0.15	0.14	0.04
33	6311359	0.00032	A	G	0.15	0.14	0.04
36	13909204	0.00034	G	C	0.14	0.14	0.04
33	2212800	0.00034	A	G	0.21	0.13	0.04
33	2220530	0.00034	A	G	0.21	0.13	0.04
13	28760300	0.00034	G	A	0.49	-0.11	0.03
38	17470175	0.00035	G	A	0.15	0.15	0.04
38	17636562	0.00035	G	A	0.15	0.15	0.04
38	17679584	0.00035	G	A	0.23	0.13	0.04
38	17970086	0.00035	G	A	0.21	0.13	0.04
13	28842831	0.00038	A	G	0.48	-0.11	0.03
13	28860446	0.00038	C	A	0.48	-0.11	0.03
13	28862074	0.00038	A	C	0.48	-0.11	0.03
13	25008163	0.00038	A	G	0.44	-0.11	0.03
13	25423168	0.00039	G	A	0.50	0.11	0.03
13	25423547	0.00039	T	A	0.50	0.11	0.03
36	9376340	0.00042	A	G	0.27	0.12	0.03
38	17874461	0.00045	A	C	0.29	0.11	0.03
13	28176188	0.00046	A	G	0.46	0.11	0.03
13	28282194	0.00046	G	A	0.46	0.11	0.03
13	25341980	0.00046	A	G	0.50	-0.10	0.03
13	25738705	0.00048	G	A	0.17	0.14	0.04
36	20496290	0.00048	A	G	0.05	0.23	0.07
36	9543177	0.00049	A	G	0.13	0.16	0.05
13	25519392	0.00049	A	G	0.44	0.11	0.03
13	28300663	0.00050	A	G	0.46	0.11	0.03
13	28974415	0.00050	G	C	0.47	0.10	0.03
13	27313776	0.00051	G	A	0.17	0.14	0.04
13	27318143	0.00051	G	A	0.17	0.14	0.04
33	5210927	0.00052	A	G	0.14	0.15	0.04
33	5222649	0.00052	G	A	0.14	0.15	0.04
33	5350151	0.00052	A	C	0.14	0.15	0.04

13	26840540	0.00054	G	A	0.49	-0.11	0.03
13	25829840	0.00054	G	A	0.46	0.11	0.03

Table S2: Details of top associated SNPs in analysis of young dogs

CHR	BP	P.value	A1	A2	Freq	b	SE
33	8104361	0.00000	G	A	0.28	0.30	0.06
33	8119663	0.00000	C	A	0.25	0.30	0.06
33	7824270	0.00001	A	G	0.28	0.28	0.06
13	25530619	0.00001	G	A	0.43	0.26	0.06
13	25423168	0.00001	A	G	0.49	-0.25	0.06
13	26152730	0.00001	A	G	0.49	-0.25	0.06
13	25423080	0.00001	G	C	0.43	0.26	0.06
13	25423547	0.00001	A	T	0.49	-0.25	0.06
13	25341980	0.00002	A	G	0.49	-0.24	0.06
13	25519392	0.00002	A	G	0.44	0.25	0.06
13	28029410	0.00003	G	A	0.42	0.25	0.06
33	7737742	0.00003	A	G	0.19	0.28	0.07
13	25829840	0.00003	G	A	0.43	0.24	0.06
13	25690710	0.00003	A	G	0.43	-0.24	0.06
13	28075353	0.00003	G	A	0.42	0.25	0.06
13	28282194	0.00003	G	A	0.42	0.25	0.06
13	26343261	0.00003	A	T	0.48	-0.23	0.06
13	25208544	0.00003	A	C	0.48	-0.23	0.06
13	25842733	0.00003	T	A	0.44	0.24	0.06
13	26230261	0.00004	A	G	0.44	-0.23	0.06
13	25346282	0.00004	A	G	0.43	-0.23	0.06
13	28223435	0.00004	A	G	0.43	0.25	0.06
13	27637942	0.00005	A	G	0.42	0.24	0.06
13	26354649	0.00005	G	A	0.48	-0.23	0.06
13	26684157	0.00006	G	A	0.44	-0.24	0.06
13	28176188	0.00007	A	G	0.43	0.24	0.06
13	28197615	0.00007	A	G	0.43	0.24	0.06
13	26935106	0.00007	A	C	0.45	-0.23	0.06
13	28300663	0.00008	A	G	0.44	0.24	0.06
13	26708681	0.00008	G	A	0.44	-0.23	0.06

Table S3. Breeds used as reference in CSS analysis with low risk of lymphoma.

Breed	Breed Group	Lymphoma Risk (OR)	Sample Size
American Hairless Terrier	American_Terrier	0.2	78
Rat Terrier		0.2	
Toy Fox Terrier		0.2	
Chihuahua	American_Toy	0.2	
Chinese Crested		0.2	
Japanese Chin	Asian_Toy	0.3	302
Lhasa Apso		0.3	
Pekingese		0.3	
Shih Tzu		0.3	
Tibetan Spaniel		0.3	
Eurasier	Eurasier	0.3	
Finnish Spitz	Finnish_Spitz	0.3	
Poodle Miniature	Poodle	0.3	
Poodle Standard		0.3	
Poodle Toy		0.3	
Standard Poodle		0.3	
Samoyed	Samoyed	0.3	
Tibetan Terrier	Tibetan_Terrier	0.3	
Tibetan mastiff	Asian_Spitz	0.5	156
Tibetan_mastiff		0.5	

Akita	0.5	
Alaskan Malamute	0.5	
Greenland Sledge Dog	0.5	
Shiba Inu	0.5	
Siberian Husky	0.5	
Xigou	0.5	

Table S4:Top genomic regions detected by Composite selection signals analysis in lymphoma affected Bullmastiff dogs.

Chromosome	Location (Mb)	Significant SNPs
1	50.91-52.86	13
1	53.17-54.95	33
1	59.73-60.85	5
3	7.81-8.97	7
3	73.1-75.63	47
3	75.93-78.85	55
5	62.3-63.9	20
7	20.22-21.42	8
7	60.9-62.17	13
8	57.85-59.48	4
9	44.58-46.33	19
10	4.94-6.39	8
10	6.59-9.47	24
18	8.23-10.41	10
18	24.84-26.36	20
20	29.92-30.89	4
22	27.83-28.96	8
26	23.67-24.64	10
30	19.25-20.64	17
32	3.73-5.64	36
